# The Mediating Role of Humor in Relation With COVID‐19 Fear, Anxiety and Depression of Bangladeshi People: A Cross‐Sectional Study

**DOI:** 10.1002/hsr2.71017

**Published:** 2025-07-09

**Authors:** Md. Shahinoor Rahman, Abdur Rahman, Murshida Ferdous Binte Habib

**Affiliations:** ^1^ Department of Psychology University of Chittagong Chattogram Bangladesh; ^2^ Department of Psychology University of Rajshahi Rajshahi Bangladesh

**Keywords:** anxiety, depression, fear of COVID‐19, sense of humor

## Abstract

**Background and Aims:**

The coronavirus (COVID‐19) outbreak induced fear and worry, which has been linked to a higher risk of mental health problems. A sense of humor may function by assisting in the reappraisal of threats or by making fear, anxiety, and depression less threatening. However, little attention was paid to its roles. Thus, this current study aims to investigate the relationship and mediating role of humor in the connection between fear of COVID‐19, anxiety, and depression.

**Methods:**

A cross‐sectional survey was conducted among Bangladeshi online users, a sample size of 628 (68.5% male) online users from different parts of Bangladesh. Their average age was 29.82 ( ± 9.67) years old. Sense of Humor Questionnaire‐6 was used to measure the sense of humor, General Anxiety Disorder‐7 scale was applied to determine anxiety, Patient Health Questionnaire‐9 was used to measure depression, and the Fear of COVID‐19 scale was used to measure the fear of the participants.

**Results:**

Sense of humor is reversely associated with fear of COVID‐19 (*r* = −0.151), anxiety (*r* = −0.226), and depression (*r* = −0.183). From mediation analysis, the model indicated that humor mediated the relationship between fear of COVID‐19, anxiety, and depression. The hypothesis model accounted for approximately 27% and 12.5% of the variance in anxiety and depression, respectively.

**Conclusion:**

This finding indicated that a greater sense of humor leads to less anxiety and depression. In another way, a sense of humor could lessen COVID‐19 fear and boost the mental health of the Bangladeshi people during this pandemic. This finding might help us understand that humor could be a protective and effective technique to fight pandemic fear. This study was only conducted in Bangladeshi culture who were literate and able to use the internet.

## 1 Introduction

The novel coronavirus‐2 (nCoV‐2) has caused the death of many people every day all around the world. Around 229 countries, including Bangladesh, have reported “700,833,973” cases of COVID‐19 and “6,962,730” deaths as of January 3, 2024 [1]. In Bangladesh, total died 29,443 out of 2,037,588 confirmed cases [2]. This number of deaths caused by the pandemic has been enormous, instilling fear in people. Fear refers to “a basic, powerful emotion elicited due to perception of impending threat, featuring an instantaneous alarm reaction that mobilizes the organism by inducing a series of physiological changes” [3]. The fear of COVID‐19 is defined as being concerned about becoming infected with the virus [4]. This concern causes a substantial psychological impact, such as anxiety and depression. Anxiety refers to a “future‐oriented emotions of tension, worrying thoughts, and bodily changes” [5], whereas depression is a mental health condition that results in intense sadness or a loss of pleasure or interest in activities over an extended period of time [6]. These mental health conditions (anxiety and depression) and fear of COVID‐19 may be influenced by sense of humor, which refers to the appreciation of humor in terms of how much and how intensely one laughs or is amused [7]. *However, no research has identified the role of sense of humor in the relationship among anxiety and depression with COVID‐19 fear. Thus, the aim of this study is to look into the relationship and mediating role of sense of humor in it*.

### 1.1 Fear of COVID‐19 and Anxiety, Depression

In the COVID‐19 pandemic, psychological problems like frustration, anger, and fear of death are rampaging in the community [8, 9]. Fear of the COVID‐19 pandemic is a natural and normal human reaction [10], as are other sudden and unexpected calamities (e.g., earthquakes and floods), as well as encounters with deadly animals. When survival circuits identify dangers, they initiate defensive behavior. Federico, Ciccarelli [11] identified the neural correlates of COVID‐19 fear and discovered a variation of major bilateral fronto‐tempo‐parietal functional brain networks. They observed selective recruitment of cortical (e.g., frontal lobes) and subcortical fear‐related regions (e.g., amygdala and putamen) of the so‐called social brain network when participants looked at COVID‐19‐related faces. So, it is not surprising that any uncertain situation or unknown disease like COVID‐19 makes people fearful [9, 12], a common source of anxiety [13] and depression.

This fear also helps individuals cope well with the situation and helps them take preventive measures [14, 15] to keep themselves safe from the threat. However, higher levels and long‐term fear have terrible consequences for physical and mental health, which leads to failure to cope well with adverse situations properly [12, 16, 17]. In addition, according to Koçak, Koçak [18], COVID‐19 fear has a stronger influence on anxiety and depression when an individual is not physically well, and when family members or friends become sick, the negative impact of that fear is exacerbated.

*Fear of COVID‐19 varies due to some factors*. Mendes, Sim‐Sim [19] conducted a study on professional caregivers for elderly patients in Portugal. Their findings revealed that women had a higher level of COVID‐19 fear, whereas those who had received the flu vaccine had a lower level of COVID‐19 fear. Their model explained 6.7% of the variation in fear of COVID‐19. *Thus, COVID‐19 fear varied according to education level, gender, and flu vaccination status*.

Tokumitsu, Sugawara [20] conducted a longitudinal study on Japanese participants and observed that while severe fear of COVID‐19 decreases significantly with therapy, mild COVID‐19 fear does not. They also found that gender (male), vaccination, higher income level, alcohol consumption, and marital status (unmarried) were all predictors of increased COVID‐19 fear. *So, a few demographics (gender, income, marriage, immunization, and alcohol) play a significant role in COVID‐19 fear, and only severe fear may be reduced through therapy*.

According to Wang, Pan [21], 53.8% of Chinese people experienced moderate to severe negative psychological problems as a result of COVID‐19, with 16.5% experiencing depressive symptoms, 28.8% experiencing anxiety disorders, and 8.1% experiencing moderate to severe stress. Similarly, in a study conducted by Huang and Zhao [22] in China, depression and anxiety were shown to be prevalent in 20.1% and 35.1% of the population, respectively. However, according to Lu, Yang [23], 23.5% of Chinese experience depression and 9.5% experience anxiety. According to the study by Guerrini, Schneider [24] in the United States, 42% of the general population experienced clinically significant anxiety, while 38% experienced clinically significant depression. *So, people in various countries have reported higher levels of depression (16.5%–38%) and anxiety (9.5%–42%)*.

Fear of COVID‐19 is associated with mental health problems such as anxiety and depression. Sharif‐Esfahani, Hoteit [25] investigated the relationship between COVID‐19 fear and anxiety as well as depression of Syrian refugees in Canada, where they found COVID‐19 fear is associated with high levels of anxiety and depression. Similarly, Khalaf, Abdalgeleel [26] found in their survey on elderly people that fear of COVID‐19 is associated with anxiety and depression. Sandín, Espinosa [27] conducted a study on coronavirus fears and transdiagnostic factors in predicting the severity of anxiety and depressive disorder in adolescents. They found that coronavirus fears, as well as other factors like negative affect, intolerance of uncertainty, acceptance/tolerance, rumination, and suppression, explained the unique variation in the severity of anxiety and depressive disorder symptoms [27]. Furthermore, Peiróa, Luque‐Garcíac [28] conducted a study on Spanish citizens and observed that COVID‐19 fear triggered somatic problems. They also demonstrated that rumination mediates the relationship between COVID‐19 fear and psychological distress, and that psychological distress leads to somatic problems. *So, relationship between COVID‐19 fear and psychological problems (somatic) is influenced by rumination*. Lu, Yang [23] found that fear of COVID‐19 was favorably connected with anxiety and depression. *So, fear of COVID‐19 link to depression/anxiety*.

*COVID‐19 fear has been linked to psychological problems such as somatic problems, anxiety, and depression in people ranging from adolescents to the elderly in various countries. Rumination and motivation are found to influence the relationship between fear of COVID‐19 and psychological problems*.

In Bangladesh, several researchers assess the psychological impact of COVID‐19 on the general population. For example, Mamun, Sakib [29] performed a nationwide survey of the general population and found that 33.3% of Bangladeshi suffered from depression, and 5% had suicidal ideation during the COVID‐19 pandemic. Similarly, Das, Hasan [30] conducted a cross‐sectional study and found that 71% of participants suffered from loneliness, 38% from depression, 64% from anxiety, and 73% from sleep disturbance. Researchers also assess the psychological consequences of COVID‐19 on home‐quarantined adults [30]. For example, Banna, Sayeed [31] evaluated the impact of mental health on home‐quarantined adults and found similar results. They identified anxiety and depressive symptoms in 33.7% and 57.9% of the adults, respectively [31]. Repon, Pakhe [32] investigated the effect of COVID‐19 on healthcare professionals and found loneliness, depression, anxiety, and sleep disturbance to be prevalent in 89%, 44%, 78%, and 87%, respectively [32]. Ashiq, Gupta [33] explored the prevalence of mental health issues and fear of COVID‐19 among medical students. They found that 51.20%, 59.40%, and 64% of students experienced moderate to severe stress, anxiety, and depression, respectively. They also observed that COVID‐19 fear was higher among students who had infected and elderly family members.

Abir, Kalimullah [34] assess the psychological impact of COVID‐19 on both general and COVID‐19 tested positive individuals. They found that approximately 64% of people had a high level of depression, 87% of people had a high level of anxiety, and 61% had a high level of stress. They also investigated that individuals who tested positive for COVID‐19 had significantly higher levels of depressive and anxiety symptoms than those who were not tested.

Kibria, Kabir [35] look at the prevalence of anxiety and depression among COVID‐19 survivors in urban areas. They found that the overall prevalence of depression and anxiety was 26.0% and 23.2%, respectively, among COVID‐19 survivors. They also found that anxiety and depression were more prevalent among elderly and hospitalized survivors than in their counterparts. Finally, Zaid, Tasnim [36] investigated the mental health status of infected COVID‐19 rural people and found that 30.9% were stressed, 21.8% were anxious, and 24% were depressed. Gender (female), low education, living without a family, living accommodations (smaller), and low economic status all significantly predicted their mental health outcomes.

*Thus*, COVID‐19 pandemic not only caused anxiety and depression worldwide, but also caused loneliness (71%–89%), sleep problem (73%–89%), stress (30.9%–61%), anxiety (21.8%–78%), and depression (24%–64%) in our country to healthcare professionals, medical students, general public, and COVID‐19 positive with home quarantine rural and urban individuals. Interestingly, the literature reviewed above indicates that COVID‐19 infected people experienced fewer mental health issues than noninfected people. It has also been observed that the prevalence of anxiety and depression is much higher in Bangladesh than in the rest of the world.

### 1.2 Sense of Humor, Anxiety, and Depression

Sense of humor would be a protector of mental health during the pandemic situation. Rnic, Dozois [37] reported that people who had less humor in their lives had higher cognitive distortion and depression. Similarly, Papousek, Aydin [38] claimed that people who had a lower tendency to laugh or were unable to laugh had a higher level of anger and showed more aggression. Besides, Fritz, Russek [39] stated that humor was a good predictor of reducing psychological distress in a stressful situation.

Qazi, Khizar [40] examined the effects of social anxiety and humor on mental health. They observed that social anxiety and humor styles have a significant impact on mental health. More specifically, they realized that adaptive humor styles, such as affiliative and self‐enhancing humor styles, reduce social anxiety and promote mental health. On the other hand, maladaptive humor, such as aggressive and self‐defeating humor, exacerbates social anxiety and causes deterioration of mental health.

Sterghos [41] investigated the effect of humor on personality and perceived stress in undergraduate students, discovering that extraversion and stress were mediated by affiliative and self‐enhancing humor. They found that affiliative humor increased stress, whereas self‐enhancing humor decreased stress. Furthermore, they discovered that neuroticism and perceived stress were mediated by self‐enhancing and self‐defeating humor. They demonstrated that self‐enhancing humor reduced stress while self‐defeating humor increased stress.

Finally, Zhao, Yin [42] reported that laughter and humor could effectively alleviate depression and anxiety, and increase sleep quality. Menéndez‐Aller, Postigo [43] identified that humor works as a protective factor against anxiety and depression. *Thus, sense of humor could defeat COVID‐19 fear and protect people from anxiety and depression in this pandemic*.

Research shows that sense of humor varies depending on gender and location. For example, Hofmann, Platt [44] observed that genders differ in terms of humor. They found that men express more aggressive humor and appreciation of humor than women. They also noticed that males are funnier than females [44]. Tsuno and Yamazaki [45] found that sense of humor orientation was only associated with sense of coherence in rural areas, not in cities. *So, the sense of humor differs in terms of gender and living area*.

### 1.3 Sense of Humor, Fear of COVID‐19, Anxiety, and Depression

Torres‐Marín, Navarro‐Carrillo [46] found that humor was related to perceiving COVID‐19 as less psychologically threatening and predicted a higher level of happiness in their structural equation model [46]. Similarly, Olah and Ford [47] examined the effect of self‐directed humor on emotional responses to COVID‐19, specifically stress and hopelessness. They found from sequential mediation analysis that self‐enhancing humor helped people perceive less stress and hopelessness associated with COVID‐19 emotional response. Self‐defeating humor, on the other hand, perceived more stress and hopelessness as a result of COVID‐19 [47]. *So, humor increases hope and decreases COVID‐19 psychological threat, and stress*.

Tsukawaki and Imura [48] investigated whether humor moderates the relationship between the extent of self‐isolation and the depressive symptoms that people experience when locked down. They found that affiliative and aggressive humor have opposite effects. Affiliative humor reduced the relationship between the extent of self‐isolation and depression, acting as a protective factor, whereas aggressive humor increased the relationship and acted as a risk factor for mental health [48].

Reizer, Munk [49] explore the role of humor in promoting well‐being during the COVID‐19 lockdown period. They found that affiliative and self‐enhancing humor had a positive and direct impact on psychological well‐being. They also identified that fear of COVID‐19 directly mediated the relationship between maladaptive humor (aggressive humor and self‐defeating humor) and well‐being, whereas the “work‐family interface” mediated the association between adaptive humor (affiliative and self‐enhancing humor) and well‐being [49].

Berro, Akel [50] investigated the role of emotion regulation in the relationship between fear of COVID‐19 and quality of life (QOL) in Lebanese adults. They found that higher levels of positive emotions were associated with higher levels of mental quality of life [50]. *So, positive emotions suppress COVID‐19 fear and increase mental quality of life. Positive emotions, such as humor, may reduce fear of COVID‐19*. Similarly, Cottingham [51] carried out a mixed‐methods study on social media among American media scholars and leaders. They claimed that humor was the most common theme on social media. Humor and fear can coexist in hyperbolic statements that make light of the outbreak. *So, humor may lessen pandemic fear*. Saricali, Satici [52] studied the influence of humor (both identified as coping techniques for dealing with stressful situations) in modulating the link between COVID‐19 anxiety and hopelessness. They showed that the effect of COVID‐19 fear on hopelessness was partially mediated by humor. They also observed higher COVID‐19 fear was linked to lower humor [52]. *So, humor influences COVID‐19 fear*.

Based on the literature reviewed above, it is possible to conclude that positive emotions such as humor increase mental quality of life, whereas, lessen COVID‐19 psychological threat; stress, hopelessness, and even COVID‐19 fear. Furthermore, humor had a partial influence on the relationship between COVID‐19 fear and its negative consequences, such as depression. Thus, humor affected the relationship between COVID‐19 fear and hopelessness.

Above all, it has been noticed that COVID‐19 fear is associated with psychological problems (somatic, stress, anxiety, depression), which is influenced by rumination and motivation. However, no research has been conducted to investigate the link or the influence of humor. Furthermore, during the COVID‐19 pandemic, humor was observed to have a positive relationship with hope and happiness, but a negative relationship with psychological threat, stress, hopelessness, and COVID‐19 fear. In addition, humor influences the relationship between COVID‐19 anxiety and hopelessness, self‐isolation and depression, and social media use and COVID‐19 fear. It is clear that humor can influence the relationship between COVID‐19 fear and psychological threats like anxiety and depression. It is also assumed that a sense of humor correlates with anxiety and depression. However, no studies were conducted during the COVID‐19 pandemic to determine the relationship between a sense of humor and fear of COVID‐19 and psychological threats such as anxiety and depression. Furthermore, no research has been conducted to directly examine the mediating effect of sense of humor on the relationship between COVID‐19 fear, anxiety, and depression in the Bangladeshi population.

Thus, the current study sought to investigate the relationship between sense of humor and COVID‐19 fear, anxiety, and depression. It also identifies the mediating role of a sense of humor in the relationship between COVID‐19 fear, anxiety, and depression in Bangladeshi inhabitants.

The specific objective of this study are (a) *How sense of humor is associated with the fear of COVID‐19, anxiety, and depression of Bangladeshi people*, (b) the *Mediating role of sense of humor in the relationship of fear of COVID‐19 and anxiety*, and finally, (c) *Mediating role of sense of humor in the relationship of fear of COVID‐19 and depression*. This finding would be helpful to understand that humor could be a protective and effective mechanism to overcome pandemic fear, like COVID‐19 fear, and the mental health problems associated with it.

## 2 Methods

### 2.1 Sample

The sample size for this study was determined using Raosoft sample size calculation [53], which takes into account a 95% confidence interval, a 5% margin of error, and a population portion of 0.5. The population of Bangladesh is approximately 16.98 million [54]. The calculator recommended 385 as the minimum study sample size. However, a total of 628 participants were included in this study; 68.5% were male and 31.5% female. Participants' average age was 30.25 ( ± 9.3) years old. Other detailed characteristics of the participants are shown in the following Table 1. Data were collected using Google Forms, and the link was shared through social media. In the beginning, 638 data were collected, but 10 data were incomplete, and 6 were extreme, so we excluded 16 data from our study.

**Table 1:** Demographic information of the participants.

Variables	Groups	*N* (%)
Gender	Male	427 (68%)
	Female	201 (32%)
Education	Secondary school	6 (1%)
	Higher secondary school	56 (8.9%)
	Bachelor	259 (41.2%)
	Master's	283 (45.1%)
	PhD	17 (2.7%)
	Others	7 (1.1%)
Residential area	Rural	141 (22.5%)
	Urban	383 (61.0%)
	Suburban	104 (16.6%)
Marital status	Married	264(42.0%)
	Unmarried	360 (57.3%)
	Others	4 (0.6%)
	Total	628

### 2.2 Instruments Used

#### 2.2.1 Bangla Patient Health Questionnaire

Participant depression was measured with the Bangla Patient Health Questionnaire (PHQ‐9), which was initially developed by Kroenke, Spitzer [55] and adapted to Bangladeshi culture by Chowdhury, Ghosh [56]. This tool is widely used to identify depressive symptoms, which is a 4‐point Likert‐type scale ranging from zero to three (0 = not at all, 1 = several days, 2 = more than half of the days, and 3 = nearly every day), and scores range from 0 to 27. The inter‐rater reliability was 0.459. Cronbach's alpha of this scale in this current study was 0.89.

#### 2.2.2 Bangla Fear of COVID‐19 Scale

The Bangla version of the Fear of COVID‐19 scale was used to measure COVID‐19 fear. This scale was initially developed by Ahorsu, Lin [12], and the Bangla version was adapted by Sakib, Bhuiyan [17]. The concurrent validity of this scale was 0.406. The test‐retest reliability was 0.87, and the Cronbach's alpha of this scale in this current study was 0.89. The tool consists of seven items with a five‐item Likert point response from 1 (strongly disagree) to 5 (strongly agree), and its score range is 7–35. The higher the score, the greater the fear of COVID‐19.

#### 2.2.3 Sense of Humor Questionnaire‐6 (SHQ)

The Bangla version of the SHQ, translated by Rahman [57], was used to assess sense of humor. The original scale had an internal consistency level of *α*= 0.85. Svebak [58] developed the original scale. The scale is composed of six items with four‐point Likert‐type responses from 1 to 4, and its score range is 6–24. The higher the score, the greater the sense of humor. The Cronbach's alpha of this scale in this study was 0.68, which Taber [59] considers to be slightly low but acceptable. The convergent validity of this scale with Style of Humor Questionnaire (affiliative/self‐enhancing) was *r* = 0.50 (*p* < 0.01).

#### 2.2.4 Generalized Anxiety Disorder (GAD‐7)

To measure anxiety, GAD‐7 was used, which was developed by Spitzer, Kroenke [60], and has good convergent validity 0.72 with the Beck Anxiety Inventory. The GAD‐7 was translated into Bangla and has good convergent validity (*r* = 0.676, *p* < 0.01) with PHQ‐9, identified by the researchers. The Cronbach α was 0.83 in this current study.

### 2.3 Procedure

Data were collected online. After preparing the questionnaire, a link was shared on social media on May 1, 2020, and data were collected up to May 30, 2020. Participants who were over the age of 18 years, had no other physical illness, had no COVID‐19‐affected patients in their family, had not yet been infected with COVID‐19, and had no experience with recent social isolation were included in this study, whereas participants who did not complete the entire questionnaire and were under the age of 18 were excluded. Before filling up the questionnaire, an informed consent form was given to each participant with a brief explanation and assured of confidentiality. There was a provision to withdraw participation in the study at any point in time. After taking their consent, participants filled up the online form in following steps. First of all, they filled up demographic information. Later, they filled up the fear of COVID‐19 and the SHQ. Finally, they completed the anxiety and depression questionnaire. A total of about 7–10 min were required to finish all the tasks.

### 2.4 Statistical Analysis

Descriptive analysis (Kurtosis and skewness) was performed to identify normality checking. Correlation analyses were used to examine the relationship among all the study variables. Based on the correlation of the variables, simple mediation analyses were performed using IBM SPSS 25.0 and macro‐program PROCESS 3.3 of Hayes and Preacher [61] for underlying relationship. For mediation analysis, bias‐corrected nonparametric percentile Bootstrap method and 95% confidence intervals (CI) have been used in this study. All tests were two‐tailed, with statistical significance established at *p* values bellow 0.05.

### 2.5 Ethical Consideration

For this study, participants provided informed consent. The researchers strongly assert that all procedures in studies involving human participants were carried out in accordance with national and institutional ethical standards, as well as the Helsinki Declaration. The researchers are completely dedicated to research ethics. The Psychology Department at the University of Chittagong provided ethical clearance, with the reference number ERB‐PSY‐CU‐55‐2021.

## 3 Results

### 3.1 The Descriptive Statistics

Table 2 shows the descriptive statistics data, and the skewness values ranged from −0.348 to 1.24, which is within Brown's [62] acceptable range of −3 to +3. Kurtosis ranged from −0.521 to 1.11, which falls within the acceptable range of −2 to +2 for a normal distribution, according to George and Mallery [63]. Gender and residence are included and controlled for in the main analysis because evidence suggests that sense of humor varies by gender [64] and residence [65].

**Table 2:** Descriptive statistics of COVID‐19 survivors (*N* = 628).

	Min	Max	M	SD	Skew	Kurt
Gender	1.00	2	—	—	—	—
Residence	1.00	2	—	—	—	—
Fear C19	7.00	34.00	19.25	5.71	0.010	−0.521
Sense of humor	10.00	24.00	19.04	2.59	−0.348	0.088
Anxiety	0.00	21.00	5.33	4.28	0.993	0.617
Depression	0.00	27.00	6.33	5.91	1.24	1.11

Abbreviation: Fear C19 = Fear of COVID‐19.

### 3.2 Correlation Analysis

Pearson's correlation values are presented in Table 3. Fear of COVID‐19 was positively correlated with general anxiety (*r* = 0.498, *p* = 0.000, *r* ^*2*^ = 0.106) and depression (*r* = 0.327, *p* = 0.000, *r* ^*2*^ = 0.106) but negatively related with sense of humor (*r* = −0.151, *p* = 0.000, *r* ^*2*^ = 0.022).

**Table 3:** Correlation among sense of humor, fear of COVID‐19, anxiety, and depression.

Variable	1	2	3	4
1. Fear of COVID‐19	1			
2. Sense of humor	−0.151*	1		
3. Anxiety	0.498*	−0.226*	1	
4. Depression	0.327*	−0.183*	0.693*	1

* Correlation is significant at the 0.05 level (two‐tailed).

### 3.3 Simple Mediation Model

**Figure 1:**
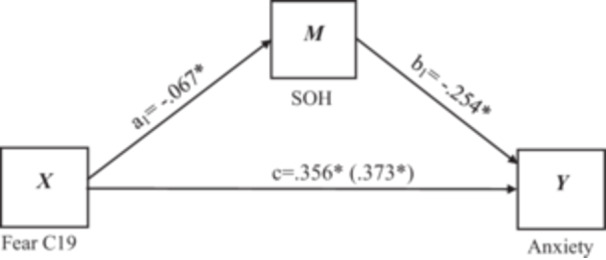
Mediating role of sense of humor in the relationship between fear of COVID‐19 and anxiety. *p < 0.05. Fear C19 = Fear of COVID‐19; SOH = sense of humor.

**Figure 2:**
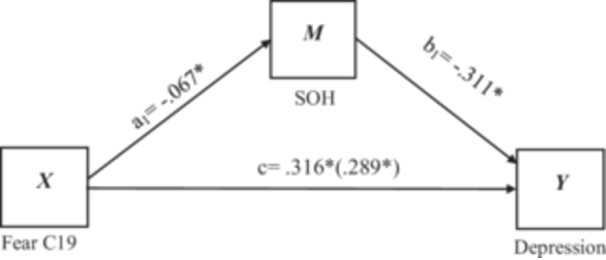
Mediating role of sense of humor in the relationship between Fear of COVID‐19 and Depression. *p < 0.05. Fear C19 = Fear of COVID‐19; SOH = sense of humor.

The possible mediating effect of sense of humor on the effect of fear of COVID‐19 on individual general anxiety and depression was tested based on the mediation test procedure of Hayes and Preacher [61] in two separate models (see Figure 1 and Figure 2). The result of the bias‐corrected nonparametric percentile Bootstrap method and 95% confidence intervals (CI) have been used (Model Number = 4, Bootstrap samples = 5000, Coding system = indicator) for mediation analysis between fear of COVID‐19 and anxiety and depression. In this model, fear was used as an independent variable, anxiety and depression were both used as dependent variables, and sense of humor, as well as controlling for gender and residence status as demographic variables. According to Hayes and Preacher [61], a substantial mediation outcome is indicated when the confidence interval for an indirect effect does not contain zero. Hayes and Preacher [61] also claimed that the bootstrap technique, which generates a sampling distribution of indirect effects, is the most powerful and valid test of mediation. Bootstrapping investigations were conducted here using Mediation Model 4 via PROCESS Macro 3.3.

#### 3.3.1 Mediation Effect of Sense of Humor on Anxiety

The mediation analysis results on anxiety are presented in Table 4. First, the result of Path c (s) showed that there was a total significant effect of fear of COVID‐19 on anxiety, *X*_1_ *b* = 0.354, *t*(3, 623) = 13.74, *p* < 0.05. Second, Path a (s) showed the direct effect of COVID‐19 fear on sense of humor was statically significant X*b* = 0.067, *t*(3, 623) = −3.79, *p* < 0.05. Third, Path b(s) showed the direct effect of sense of humor on anxiety, Y *b* = −0.254, *t*(3, 623) = −4.59, *p* < 0.05. The Path c' showed that when sense of humor (mediator) was entered into the model, the relationship between fear of COVID‐19 and anxiety changed significantly, X_1_ *b* = 0.373, *t*(3, 623) = 14.36, *p* < 0.05. These outcomes accept the mediation hypothesis. The model is significant, *F* (4, 622) = 58.25, *p* < 0.05, and explained approximately 27% (*R* ^2^ = 0.2725) of the variance in anxiety.

**Table 4:** Mediation analysis of sense of humor on the effect of fear of COVID‐19 on anxiety.

Model pathway		Coefficient	SE	*t*	*p*	95% Confidence interval	
						Lower	Upper
a path	Fear C19 → SOH	−0.067	0.179	−3.79	0.0001	−0.1035	−0.0329
b path	SOH → GAD	−0.254	0.001	−4.59	0.0000	−0.3664	−0.1424
c path	Fear C19 → GAD	0.356	0.025	13.74	0.0000	0.3051	0.4068
ć path	Fear C19 → GAD	0.373	0.026	14.36	0.0000	0.3223	0.4243

Abbreviations: GAD = General Anxiety Disorder; SOH = sense of humor.

##### 3.3.1.1 Indirect Effect of Sense of Humor on Anxiety

The comparison of direct and specific indirect effects of fear of COVID‐19 on anxiety through sense of humor. The results were presented in Table 5. The indirect effect was verified by 5000 bootstrap samples. The 95% confidence interval of the simple mediation model excluded zero [0.0062, 0.0323], suggesting a significant mediating effect of sense of humor on the relationship between fear of COVID‐19 and general anxiety, X indirect *=* 0.0173, *SE* = 0.006. According to Hayes and Preacher [61], when the confidence interval for an indirect effect does not contain zero, it indicates a significant mediation outcome.

**Table 5:** The comparison of direct and indirect effects of fear of COVID‐19 on anxiety.

Product of coefficients						95% confidence interval	
		Estimate	SE	*t* value	*p*	Lower	Upper
Total effect	Fear C19 → GAD	0.373	0.026	14.36	0.0000	0.3223	0.4243
Direct effect	Fear C19 → GAD	0.356	0.025	13.74	0.0000	0.3051	0.4068
Indirect effect	Fear C19 → SOH → GAD	0.017	0.006			0.0060	0.0322

*Note: N* = 628, Control Variable: Gender, residence.

#### 3.3.2 Mediation Effect of Sense of Humor on Depression

The mediation analysis results on depression were presented in Table 6. First of all, the result of Path c (s) showed that there was a total significant effect of fear of COVID‐19 on depression, X_1_ *b* = 0.316, *t*(3, 623) = 8.07, *p* < 0.05. Secondly, Path a (s) showed the direct effect of COVID‐19 fear on sense of humor was statically significant X*b* = 0.067, *t*(3, 623) = −3.79, *p* < 0.05. Thirdly, Path b(s) showed the direct effect of sense of humor on depression, Y *b* = −0.311, *t*(3, 623) = −3.60, *p* < 0.05. The Path c' showed that, when sense of humor (mediator) was entered into the model, the relationship between fear of COVID‐19 and depression changed significantly, X_1_ *b* = 0.338, *t*(3, 623) = 8.63, *p* < 0.05. These outcomes accept the mediation hypothesis. The model is significant, *F* (4, 622) = 22.34, *p* < 0.05, and explains approximately 12.5% (*R* ^2^ = 0.1256) of the variance in depression.

**Table 6:** Mediation analysis of sense of humor on the effect of fear of COVID‐19 on depression.

Model Pathway		Coefficient	SE	*t*	*p*	95% Confidence interval	
						Lower	Upper
a path	Fear C19 → SOH	−0.067	0.179	−3.79	0.0001	−0.1035	−0.0329
b path	SOH → DEP	−0.311	0.086	−3.60	0.0004	−0.4805	−0.1408
c path	Fear C19 → DEP	0.316	0.039	8.07	0.0000	0.2399	0.3940
ć path	Fear C19 → DEP	0.338	0.039	8.63	0.0000	0.2613	0.4151

Abbreviations: DEP = Depression; SOH = sense of humor.

##### 3.3.2.1 Indirect Effect of Sense of Humor on Depression

A comparison between direct and specific Fear of COVID‐19 has an indirect effect on depression via sense of humor. The results were presented in Table 7. The indirect effect was verified by 5000 bootstrap samples. The 95% confidence interval of the simple mediation model excluded zero [0.0062, 0.0412], suggesting a significant mediating effect of sense of humor on the relationship between fear of COVID‐19 and general anxiety, X indirect *=* 0.021, *SE* = 0.009.

**Table 7:** The comparison of direct and indirect effects of fear of COVID‐19 on depression.

Product of coefficients						95% confidence interval	
		Estimate	SE	*t* value	*p*	Lower	Upper
Total effect	Fear C19 → DEP	0.338	0.039	8.63	0.0000	0.2613	0.4151
Direct effect	Fear C19 → DEP	0.316	0.039	8.07	0.0000	0.2399	0.3940
Indirect effect	Fear C19 → SOH → DEP	0.021	0.009			0.0062	0.0412

*Note: N* = 628, Control Variable: Gender, residence.

## 4 Discussion

The aim of this study was to investigate whether there was any relationship of humor with fear of COVID‐19, anxiety, and depression, and the mediation effect of sense of humor on the relationship of fear of COVID‐19, anxiety, and depression. We found from our results that sense of humor was negatively correlated to fear of COVID‐19, anxiety, and depression. In other words, the increase of sense of humor decreases the fear of COVID‐19, anxiety, and depression. These findings were consistent with the findings of Abril, Szczypka [66], Qazi, Khizar [40], and Zhao, Yin [42], which showed that humor related to a fear control response [66] and negatively related to social anxiety [40] and depression [42]. A possible explanation of this finding would be that the universal trigger of fear is the threat of harm, which protects us from danger. Those who have a high sense of humor can cope well with fear in adverse situations [67]. Moreover, According to the benign violation theory, fear of COVID‐19 is an implicit assessment of threat; all that is required for something frightening to become amusing for is a concurrent assessment of benignity. Fear and humorous amusement progression occur in the same brain structure (amygdala), but have completely opposite neurochemical profiles [68]. Thus, the antagonistic relationship between fear makes sense of humor ideal for managing coping with fear in COVID‐19 pandemic, as well as the cascade of stress hormones, including adrenaline, noradrenaline, and cortisol [69]. As a result, increasing one's sense of humor reduces anxiety and depression. Our findings are slightly inconsistent with those of Autenrieth, Asselmann [70], who showed that fear of COVID‐19 was not initially connected with anxiety symptoms but rather with health anxiety throughout the pandemic.

The further two structural equation models of mediation showed a clear significant sense of humor effect on the relationship between COVID‐19 fear and anxiety, and depression separately. In the first model, sense of humor significantly mediates the relationship between COVID‐19 fear and anxiety. In other words, the sense of humor significantly influences the relationship between COVID‐19 fear and anxiety. This finding is consistent with the study of Abel [71], and Hirosaki, Ohira [72], who indicated that humor could play a significant role in preventing negative consequences of persisting anxiety during the pandemic situation.

Likewise, our first model, the second mediation model, also provided clear evidence that sense of humor also significantly influences the relationship between COVID‐19 fear and depression. This finding was consistent with the findings of Fritz, Russek [39], Zhao, Yin [42], and Saricali, Satici [52]. In their studies, they showed that humor plays a significant role in the relationship between stressful life events and psychological distress, and humor significantly influences depression, and humor influences COVID‐19 fear and hopelessness. In contrast, our findings are inconsistent with those of Tsukawaki and Imura [48], who observed that aggressive humor increased the risk factor for mental health. Our findings suggested that people who tend to laugh could also handle depression successfully. In other words, sense of humor successfully beat COVID‐19 fear and depression.

The biological explanation of our findings is as follows. On one hand, COVID‐19‐like threatening situations may stimulate the amygdala, resulting in the release of norepinephrine, which is thought to be responsible for fear [15, 73]. Fear may alter the large gray matter volume in the amygdala, which is consistently found in General Anxiety Disorder patients [74]. Furthermore, a higher volume of the amygdala, ventral cingulate, and dorsomedial prefrontal cortex are also responsible for anxiety and depression [75]. On the other hand, those with a greater sense of humor are more likely to laugh than those with a poor sense of humor, and laughing suppresses the bioactivities of epinephrine, cortisol, and 3,4‐dihydrophenylacetic acid. Reduced neurotransmitter activity, especially norepinephrine, serotonin, and dopamine, is associated with mental health (anxiety and depression) [76, 77]. Thus, the relationship among COVID‐19 fear and mental health (i.e., anxiety and depression) is influenced by the sense of humor in our study.

## 5 Recommendation

This study's findings have important implications for teachers, practitioners, and legislators. *First*, this study highlighted the importance of pandemic‐related fear. This form of fear has a harmful impact on students' mental health. It is suggested that authorities take suitable steps to fortify protective measures among students by using humor rather than creating a tense environment, which can have an impact not only on their psychological health but also on their academic performance. *Second*, developing programs that encourage the use of humor during lockdowns can improve the mental health outcomes of the general population. *Third*, well‐established humor‐based interventions may be introduced to reduce the negative impact of COVID‐19 fear on vulnerable individuals (i.e., health professionals). *Finally*, policymakers may incorporate humorous activities in future pandemic‐like situations to address pandemic dread in their policies.

### 5.1 Limitations and Future Direction

There are several limitations in the study. *First of all*, there can be a potential selection bias in online surveys. Senior citizens and pregnant women, who were more vulnerable to the SARS‐CoV‐2 virus and were at greater risk of mental and physical suffering, were not included properly. *Second*, in this study, only those persons were included who were literate and able to use the Internet. Thus, illiterate and non‐internet users were not included in this study. *Furthermore*, we did not consider the influence of the participants' education levels and religious beliefs in this study. *Next*, the participant's social network and social support were not considered in this study. *Finally*, the study's gender imbalance and cross‐sectional survey were two of its limitations. In the future, one may observe the short or long‐term effects of humor on COVID‐19 fear and its consequences for mental health, including vulnerable people (aged and pregnant), illiterates, non‐internet users, and those lacking social support with equal gender.

## 6 Conclusion

In conclusion, in the present study, it was found that the sense of humor is inversely related to fear of COVID‐19, anxiety, and depression. In addition, a sense of humor successfully mediated the relationship between COVID‐19 fear and mental health (anxiety and depression). Thus, this study explored the mediating role of sense of humor on mental health during the COVID‐19 pandemic, which had previously been overlooked by other researchers. So, it can be said that a sense of humor can protect against fear and poor mental health during pandemic‐like situations.

## Author Contributions

**Md. Shahinoor Rahman:** conceptualization, methodology, visualization, writing – original draft, software, project administration, writing – review and editing. **Abdur Rahman:** data curation, formal analysis, investigation, validation. **Murshida Ferdous Binte Habib:** supervision, writing – review and editing, resources.

## Conflicts of Interest

The authors declare no conflicts of interest.

## Transparency Statement

The lead author Md. Shahinoor Rahman affirms that this manuscript is an honest, accurate, and transparent account of the study being reported; that no important aspects of the study have been omitted; and that any discrepancies from the study as planned (and, if relevant, registered) have been explained.

## Data Availability

The data used in this paper are available from the author upon reasonable request that follows institutional guidelines.
